# The impact of environmental change on landslides, fatal landslides, and their triggers in Pakistan (2003–2019)

**DOI:** 10.1007/s11356-022-24291-z

**Published:** 2022-12-10

**Authors:** Waqas Shabbir, Talha Omer, Jürgen Pilz

**Affiliations:** 1 Institut Für Statistik, Alpen Adria Universität Klagenfurt, Universitätsstraße 65-67, Klagenfurt Am Wörthersee, Kärnten 9020 Austria; 2 Department of Economics, Finance and Statistics, Jönköping International Business School, Jönköping University, Jönköping, 551 11 Sweden

**Keywords:** Landslides, Debris flow, Fatal landslides, Triggers, Environment, Geography, Pakistan

## Abstract

The actual impact of landslides in Pakistan is highly underestimated and has not been addressed to its full extent. This study focuses on the impact which landslides had in the last 17 years, with focus on mortality, gender of deceased, main triggers (landslides and fatal landslides), and regional identification of the hotspots in Pakistan. Our study identified 1089 landslides (including rockfalls, rockslides, mudslides, mudflows, debris flows) out of which 180 landslides were fatal and claimed lives of 1072 people. We found that rain (rainfall and heavy rainfall)-related landslides were the deadliest over the entire study period. The main trigger of landslides in Pakistan is heavy rainfall which comprises over 50% of the triggers for the landslide, and combined with normal rainfall, this rate climbs to over 63%. The second main reason for landslide occurrence is spontaneous (due to rock instability, erosion, climate change, and other geological elements) with landslides accounting for 22.3% of all the landslides. Landslides caused by rain-related events amounted to 41.67% of the fatalities, whereas spontaneous landslides caused 29.44% of the deaths and the human induced events accounted for 25.5% of the fatalities. The fatal landslides accounted for 19.53% deaths of the children. Our study also found that more than 48% of the deadly landslides occurred between the months of January to April, whereas the least fatal landslides occurred in the month of June which accounted for only 3% of all the fatal landslides in Pakistan.

## Introduction

One of nature’s most deadly disasters is a landslide, defined as the motion of the earth down the slope, rockslide, rockfall, or debris flow (USGS 2019; Hungr et al. 2014). Actual landslides are far more dangerous than most people are aware of. Landslides occur due to various reasons that can include environmental factors such as rock decay over time, and rainfall and flash floods can trigger rockfalls and debris flow. Similarly, constructions along the banks of slopes can make the slope weak, and hence, an Earth mass (a combination of mud and rock) can slide downhill, or it can be triggered by natural phenomena like earthquakes (Polemio and Petrucci 2000; Guzzetti et al. 2008; Lacroix 2016; Bradley et al. 2019).

Landslides cause thousands of deaths and injuries every year along with heavy economic losses (both directly and indirectly) by destroying infrastructure, properties, businesses, crop lands, highways, and commute roads (Wu et al. 2014; Niu et al. 2014; Del-Soldato et al. 2019). Karakoram Highway (KH hereafter) is the main highway that observes the majority shares of the trade between China and Pakistan while it is also highly active in terms of landslides and rockfalls, and there are some areas on that highway which are more susceptible to landslides than other ones (Ali et al. 2018; Zhao et al. 2019; Khan et al. 2019; Huang et al. 2019). There is a high correlation between the landslides and slope failures of the mountains or hills, and such landslides pose greater threats to the valleys as the vulnerability of mountain slopes gets higher due to weathering, deforestation, and other human-induced activities (Cerri et al. 2017; Skilodimou e al. 2018; Huang et al. 2019; Schönfeldt et al. 2022).

Pakistan is prone to earthquakes, and the greatest number of earthquakes is reported from the northern Pakistan which not surprisingly sees the greatest number of landslides (Kiani et al. 2022). Prolonged rainfalls in the monsoon season bring along dangers with them as rains have been identified as a major trigger for triggering the landslides in general (Iverson 2000; Gariano and Guzzetti 2016). Heavy precipitation is the main trigger for the landslides in several of the studies done worldwide, and the case is similar in Pakistan as well, which sees its highest amount of rainfall in the Monsoon season (Keefer et al. 1987; Gerrard and Gardner 2000; Naranjo 2007; Lazzari and Piccarreta 2018; Omer et al. 2019, 2022). A study on the effects of fatal landslides on the global scale also validates that the extreme rainfall is the major cause of landslides in more than half of the cases of landslides triggered by rainfall (Haque et al. 2019). There is also a large amount of literature available that studies the relationships between landslides and earthquakes (Tibaldi et al. 1995; Bozzano et al. 2011; Zhu et al. 2013; Parker et al. 2017; Tian et al. 2019).

Construction and disturbance along the slopes are another main factors that can generate landslides (Arbanas and Dugonjic 2010; Froude and Petley 2018; Tanyaş et al. 2022; Zevgolis et al. 2019; Zhu et al 2013). A heavy landslide, in January 2010, had completely blocked the Hunza River, and it formed an artificial lake at Attabad. It swallowed at least 3 villages, and thousands of people had to flee, and the effects of such a huge landslide were far reaching as it had also taken a large part of KH under water (Cook and Butz 2013). An alternative route was soon selected, and tunneling got started which also made the situation worse as the slopes became more unstable due to blasting of the mountains around Gojal and Attabad in Hunza valley, Baltistan.

Our study is focused at identifying the hotspots for landslides (fatal and nonfatal) in Pakistan and studying the main triggers behind these events in a 17-year timeline. The study period is divided in two sub-sections, 2003–2010 and 2011–2019, to study the trigger change effects, if there are any. We also focus on the gender of the deceased along with distribution of children, elderly and labor (note that we could only identify gender of 32% fatalities in the period 2003–2010 and 50% in 2011–2019). Furthermore, we study the dynamics of the trigger and the destructions it caused on a yearly basis and the distribution of landslides on a monthly basis.

## Data and methodology

### Data collection

The data for this study comprises 17 years, monthly based data, and the data was collected through various online publishing sources. We created an updated catalog of the data for all the landslides in Pakistan from 2003 to 2019. The main sources for the data include NASA global landslide catalog, ArcGIS landslide database, Pamir Times, Dawn News, The News International, ReliefWeb (NASA GLC, Pamir Times, ReliefWeb, Dawn News, The News, ArcGIS Data). There were a total of 1089 landslides recorded in the 17-year period with 180 being fatal landslides which claimed lives of 1072 people (note that this study excludes the event of earthquakes in October 2005 due to unreliability of the regional casualties occurred exclusively due to landslides event in northern Pakistan and Azad Jammu and Kashmir (AJK)).

**Fig. 1:**
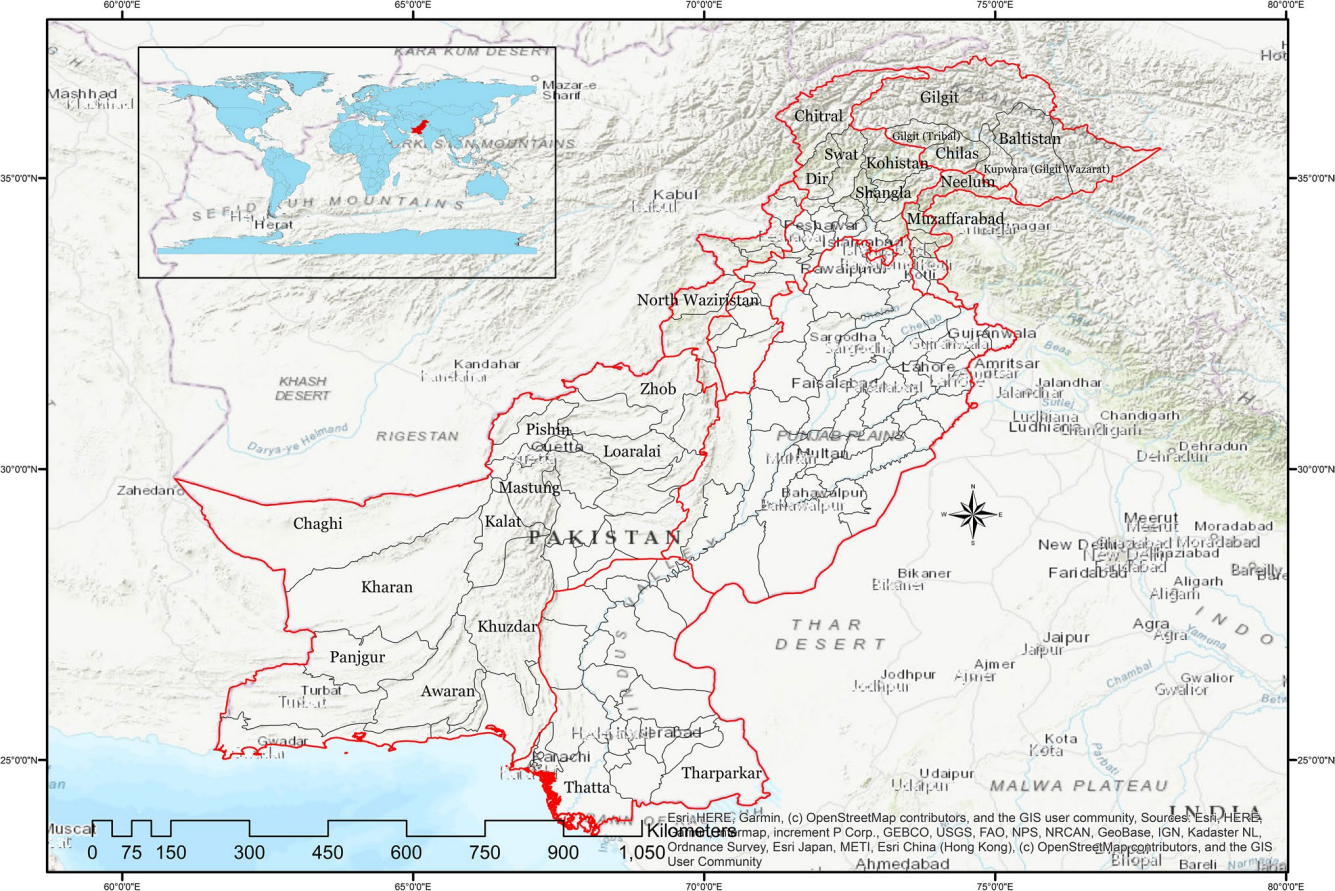
Study area for landslide investigations across Pakistan 2003–2019

This study focuses on the number of landslides (land movement, landmass fall, rockfall, rockslide, debris flow etc.) and their main triggers. It also emphasizes on their impacts (loss of life, injury, loss of property, vehicles, crops, business, etc.) in the districts all over Pakistan including the Gilgit-Baltistan region and AJK (Fig. 1). There is also enough evidence (according to residents of the northern region) that a high number of landslides and rockfalls are not even reported because of their small scale or remoteness of the regions, and people are so much used to such events that the small-scale landslides or rockfalls are cleared from the roads and it does not get recorded. However, our understanding is that a full database can help scientists and researchers to study the landslide phenomena in a more effective way.

### Methodology

**Fig. 2:**
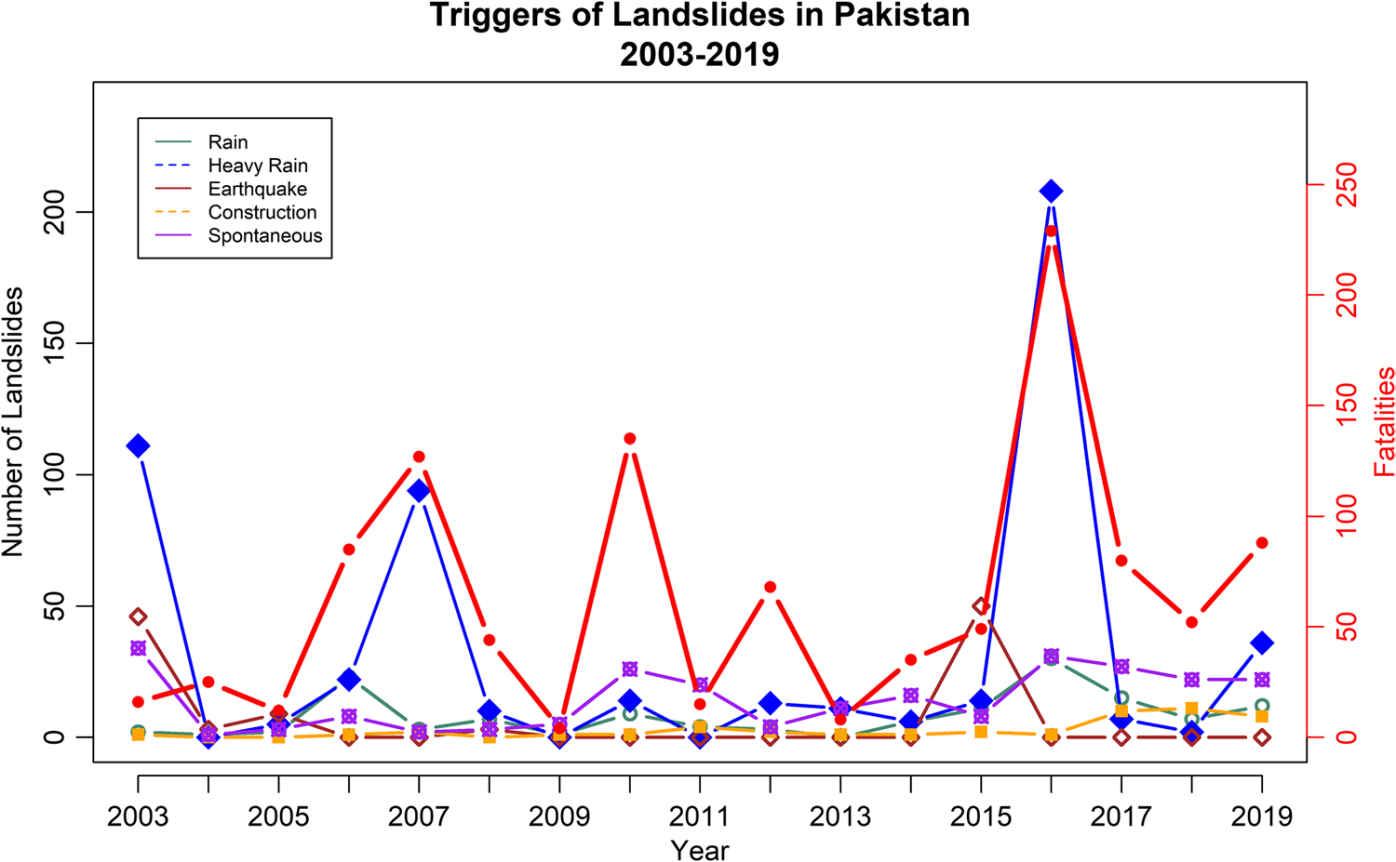
Frequency of main triggers of landslides in Pakistan 2003–2019

**Fig. 3:**
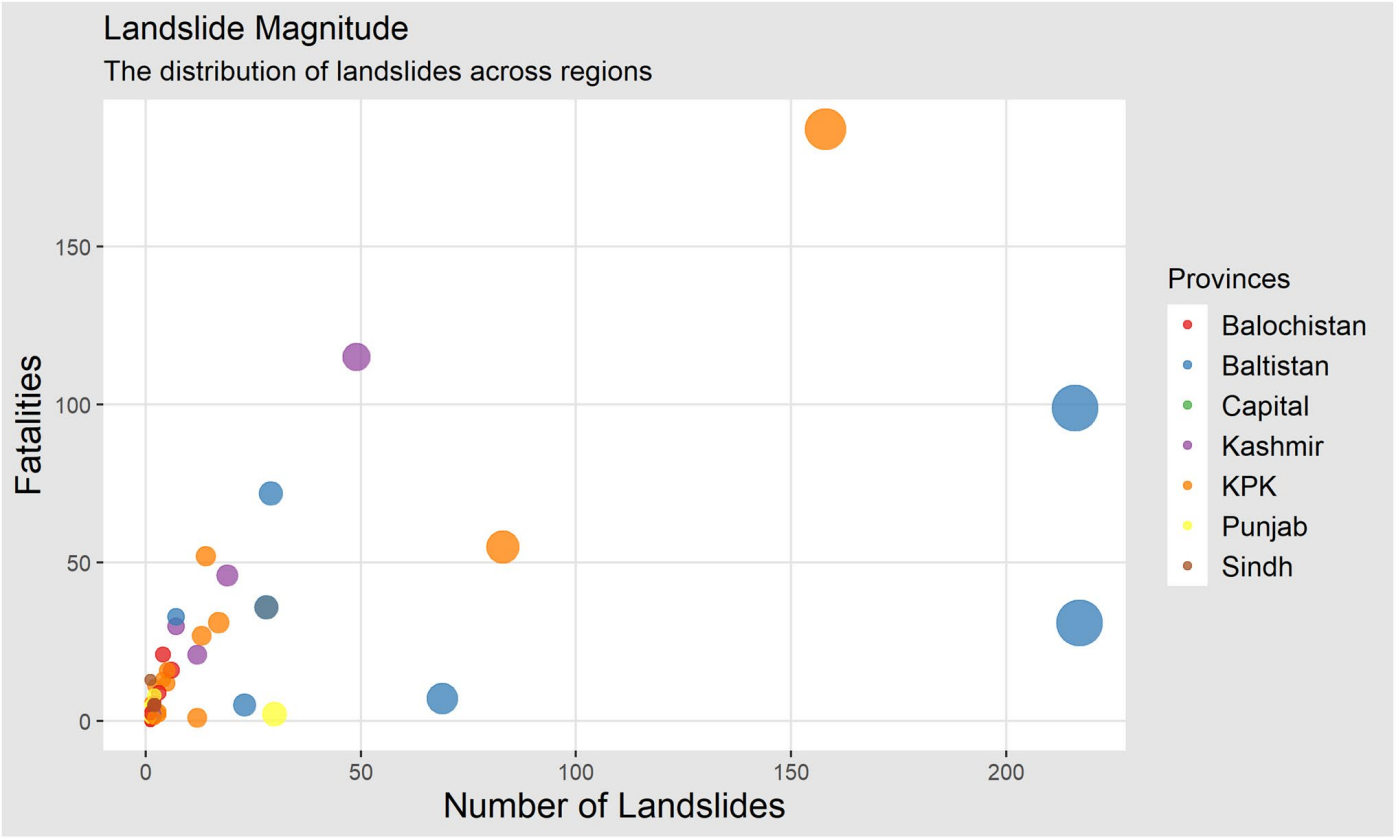
Landslide magnitude dispersion among provinces of Pakistan 2003–2019

**Fig. 4:**
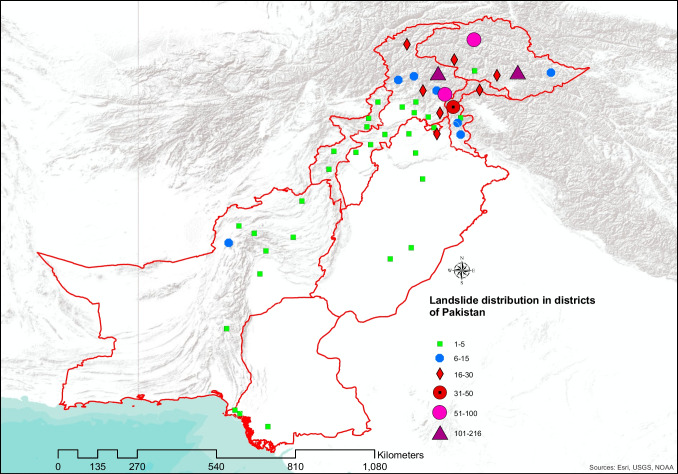
Landslides recorded in all the districts of Pakistan (2003–2019)

**Fig. 5:**
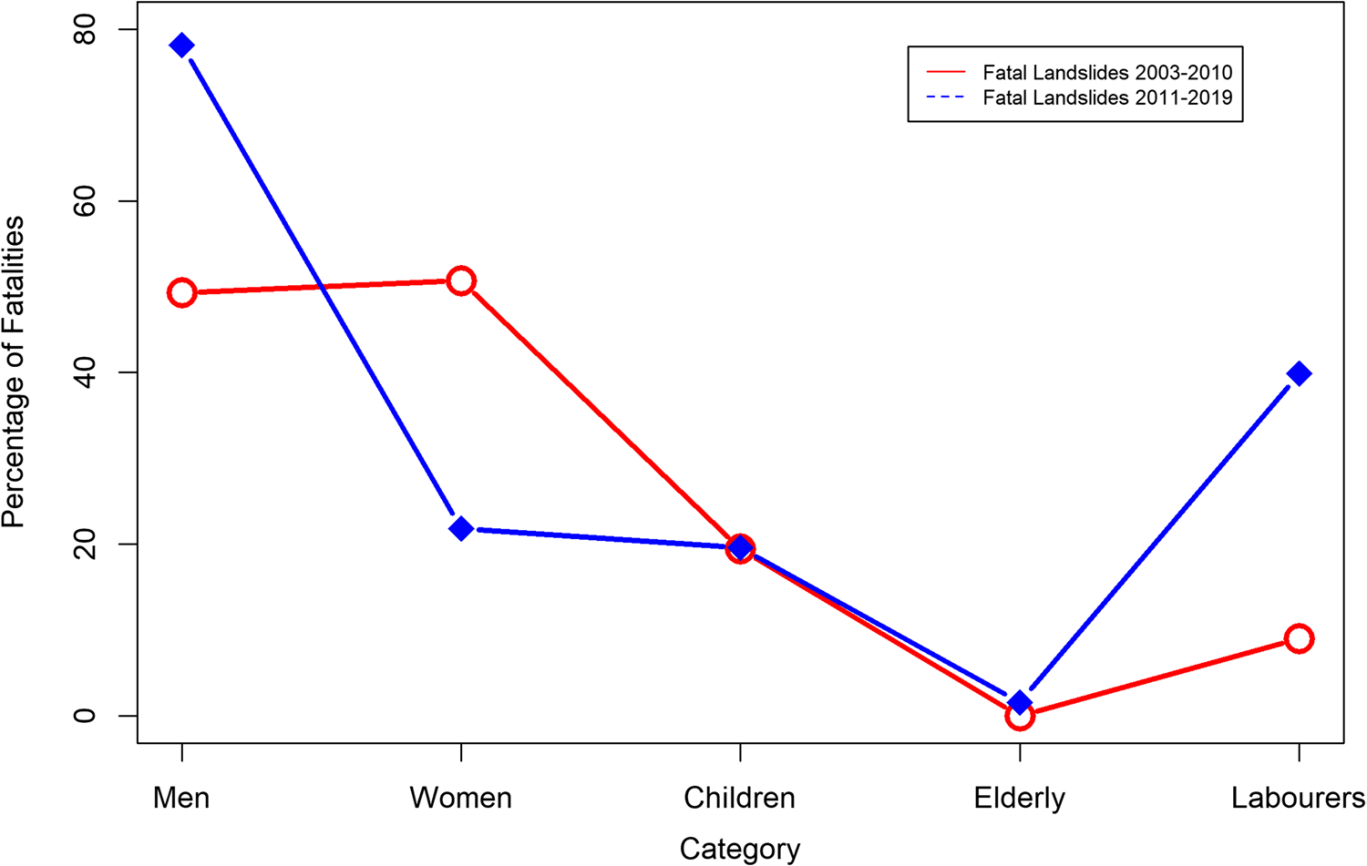
Percentage of fatalities among gender, elderly, and workers

**Fig. 6:**
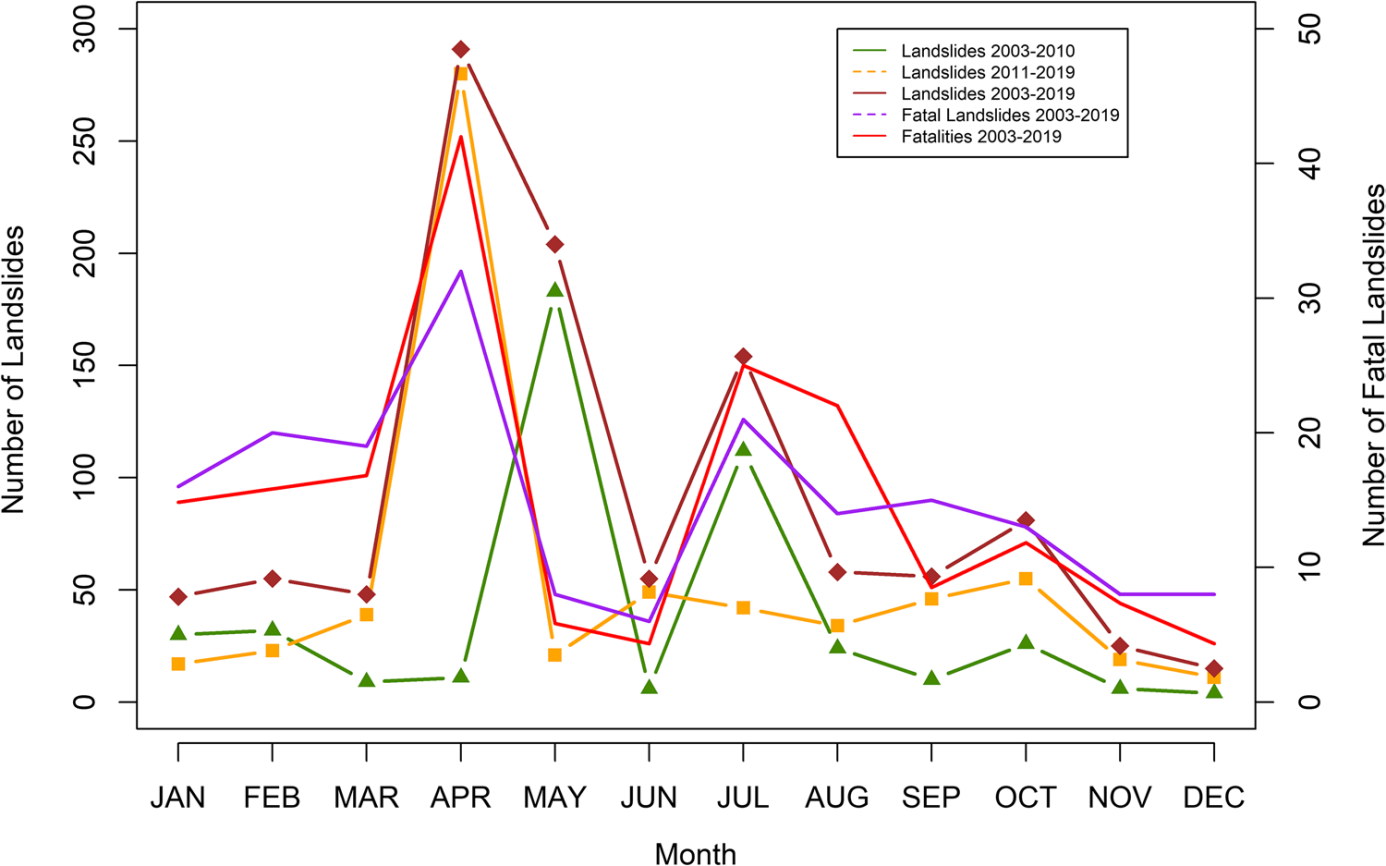
Monthly frequency of landslides and fatal landslides

**Fig. 7:**
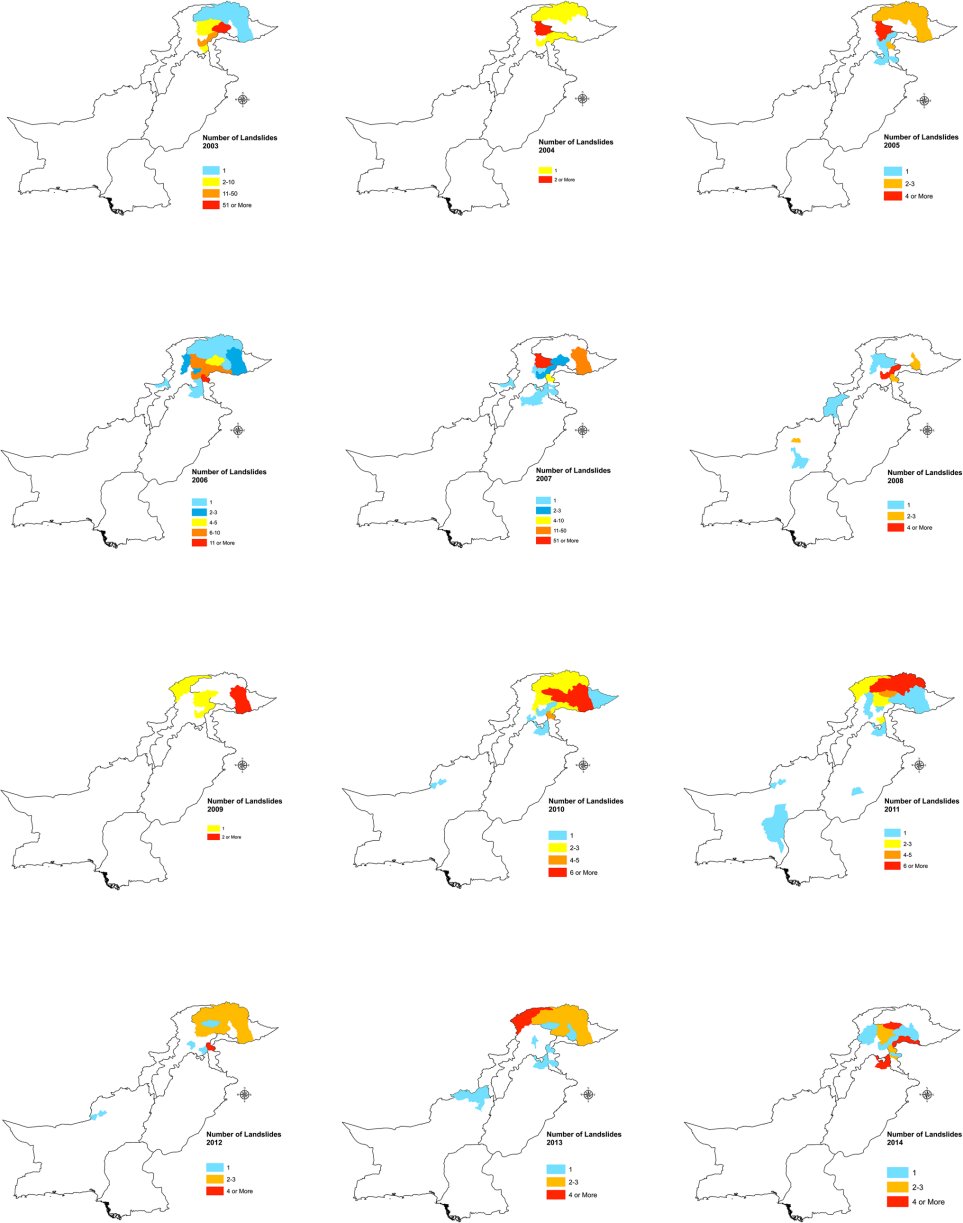

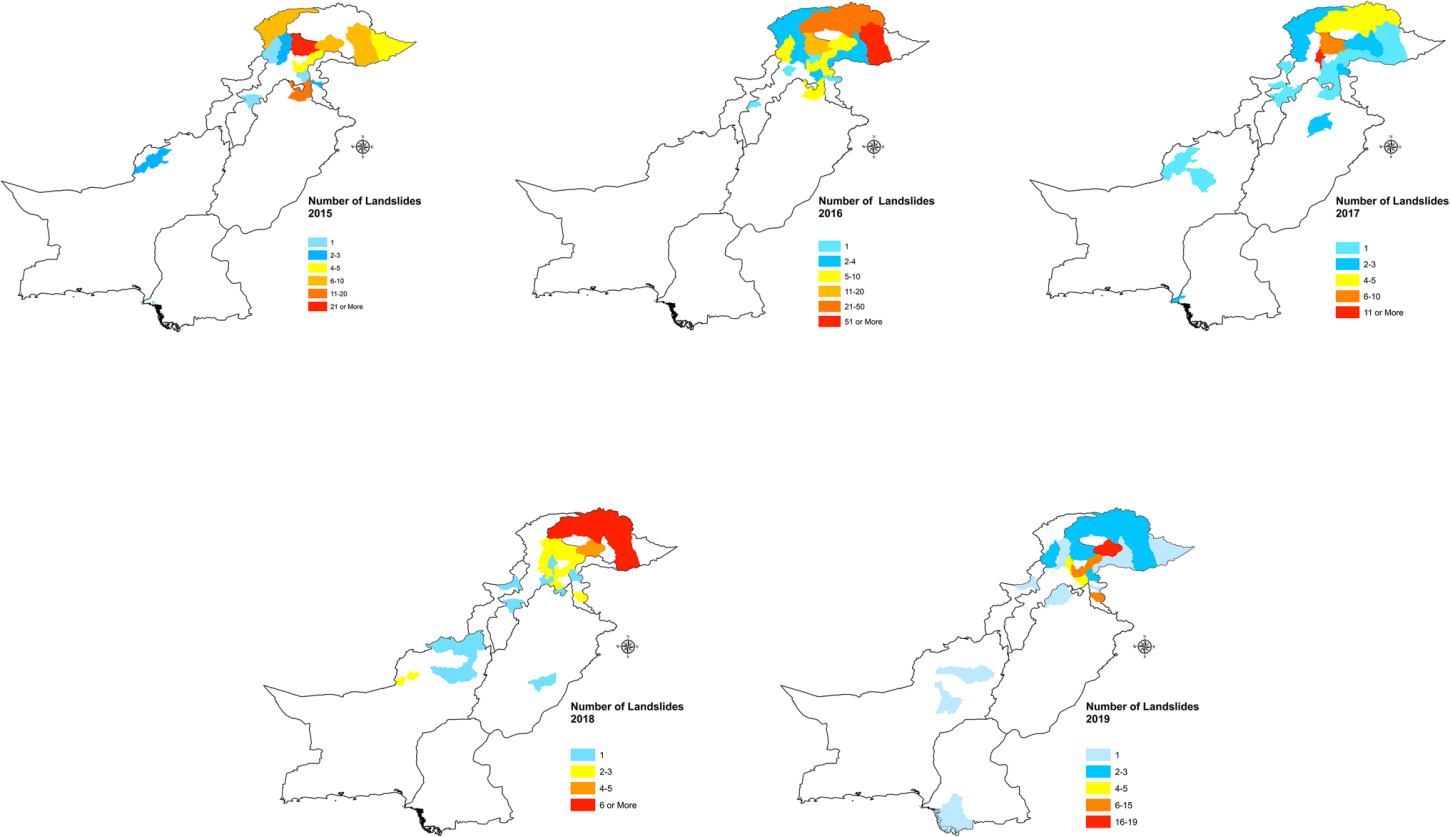
**a**–**c** Landslide hotspots in the districts of Pakistan (2003–2019)

We have analyzed the impacts of landslides in Pakistan by using the percentage analysis, and we have used ArcGIS 10.5 for the maps to analyze the concentration/hotspots of the landslides in the districts included in our study (Figs. 2, 3, 4, 5, 6, and 7a–c). R-Studio is used to make the graphical assessment of the landslides data based on 204 weeks (Fig. 2).

## Analysis and results

The data based on 17 years was segregated into two portions, 2003–2010 and 2011–2019, to examine the behaviors of the landslide triggers in spatio-temporal terms. Table 1 shows the frequency of the landslide events recorded yearly along with the casualties (dead and injured) and the frequencies of landslide triggers (rainfall, heavy/extreme rainfall, earthquakes, construction/mining, and spontaneous).

**Table 1:** Number of landslides along with recorded casualties and their triggers 2003–2019

						Triggers		
Year	Number of landslides	Fatalities	Injuries	Rainfall	Heavy rainfall	Earthquakes	Construction	Random
2003	194	16	9	2	111	46	1	34
2004	5	25	6	1	0	3	0	1
2005	19	12	0	2	5	9	0	3
2006	54	85	40	23	22	0	1	8
2007	101	127	45	3	94	0	2	2
2008	23	44	14	7	10	3	0	3
2009	7	4	3	1	0	0	1	5
2010	50	135	126	9	14	0	1	26
2011	28	15	9	4	0	0	4	20
2012	22	68	25	3	13	0	2	4
2013	23	8	3	0	11	0	1	11
2014	29	35	37	6	6	0	1	16
2015	85	49	14	11	14	50	2	8
2016	270	229	41	30	208	0	1	31
2017	59	80	45	15	7	0	10	27
2018	42	52	40	7	2	0	11	22
2019	78	88	48	12	36	0	8	22

Most landslides were recorded in 2016 with the highest number of fatalities that account for 21.4% of all the fatalities in our study period. Rainfall-related events were the major cause of deaths, and it accounted for 41.67% of the total deaths caused by the landslides in our 17-year period. In March of 2016, Pakistan received 77.4% above normal rainfall, causing heavy flood damages in at least 53 districts, and most of the damage occurred in the province of Khyber Pakhtunkhwa (Qaiser 2016). Interestingly, 29.44% of the fatal landslides were triggered spontaneously (which could be attributed toward unstable rocks, deforestation etc.). The rainfall was responsible only for 32.23% of fatal landslides from 2011 to 2019 as compared to 61% from 2003 to 2010. The majority of fatal landslides in the last decade were caused by construction events (road construction, digging, mining along steep hills and mountains) and spontaneous events that account for 66.1% of the fatalities from 2011 to 2019.

Karakoram Highway (KH) is the main link that joins all the regions of Gilgit-Baltistan with the rest of Pakistan, and it is especially important because most of the trade between China and Pakistan is happening from this land route. Billions of dollars of goods and services pass through KH every year, and it is highly vulnerable to landslides. The highway is a hotspot for the landslides and rockfall due to several reasons including continuous expansion of KH. Most of the landslides are recorded in the rainfall seasons (monsoon) which causes movement of the lose rocks and debris on the slopes. There is only a small portion where the highway is being protected by a cemented wall of rock, barely few feet high, and in practice, this is only effective in cases of small rockfalls but falling of heavy boulders are a common occurrence.

Pakistan has 154 districts, and landslides in the last 17 years are reported from 48 districts, among which 28 districts only recorded one or two landslides. The remaining 20 districts recorded larger numbers of landslide events. Baltistan and Diamer districts were responsible for 39.58% of all the landslides in our study period. The months of April through June are the time when such events are occurring most often, and it has triggered 50.5% of all the landslides in our study duration. However, it was not the deadliest since it accounted for 25.54% of the deaths, whereas the first quarter of the year is responsible for 30.54% of all the deaths in 17 years. In our study period, October through December accounts for the least number of landslides events with just 11.11% of the total landslide occurrences.

The Wilcoxon-Mann–Whitney (WMW) test was performed on the fatal landslides of the two data sets and it found that there is significant increase in the occurrences of fatal landslides in the period 2011–2019 compared to 2003–2010. The WMW test is a commonly used method to compare two conducts when the rudimentary distribution of the outcome variable is not normally distributed (Qu et al. 2008). The magnitude of the recorded number of occurrences of the landslides in all the districts of Pakistan shows that there is large clustering in the Northern regions that include Gilgit-Baltistan and regions of AJK most notably in Muzaffarabad and Neelam valley (Fig. 4).

The gender proportion that had sustained casualties was challenging because of the unavailability of credible information. In our study period from 2003 to 2010, only 32% of the gender of fatalities could be verified, and based on these statistics, 49% of the fatalities were men, and 51% of fatalities were from women, while 19.44% were children. During the period from 2011 to 2019, the fatalities among men were more than 78%, while 22% of deceased were women; these statistics are based on 50% of the known identity of the gender in the fatal landslides during this period. In the second part of our study period, 39.87% of fatalities occurred due to construction (labor, mining, expansion, etc.), whereas 19.62% were children among the fatalities from 2011 to 2019 (Fig. 5).

There is a large difference between the two periods of our study while studying the gender proportions mainly because in the later part of study where construction and spontaneous landslide events were involved, mostly men are doing the jobs of labor on the roads sides and driving the vehicles to transport goods and who were hit with spontaneous boulders and landslides.

The landslides occurrences according to the monthly basis was analyzed, and it showed that April is the month with the greatest number of landslides occurrences, and it also was responsible for the largest number of fatal landslides, 32 fatal landslides, which claimed lives of over 250 people (Fig. 6). One similarity that can be found is that large numbers of landslides are occurring during the second quarter of the year or at the start of summer season. It is observed that during the snow melting period, the vulnerable slopes give weight and lose rocks and also get help from precipitation or heavy precipitation events, for example, cloud bursts or typhoons (Fig. 6).

The damages were also accessed that can be attributed to landslides (both directly and indirectly). In the time period from 2003 to 2010, 576 homes were damaged or destroyed, and at least 20 businesses were destroyed due to landslide events. The agricultural land effects were also considered, and 10 separate events of crop destruction were recorded, and at least 10 vehicles were damaged or destroyed in such landslide events. As many as 234 roadblocks were recorded, and 13 roads (roads and small bridges) were destroyed. In the second period, 2011–2019, 489 homes were damaged or destroyed, 87 businesses were lost, at least 5 events of crop damages or destruction were observed, 34 vehicles were destroyed, 745 roads (main roads and link roads) were blocked, and 14 roads were destroyed in landslide-related events. These damages are estimated to be in the multiple of millions of dollars, and in a country that is not renowned for its economic superiority, landslides are indeed costly disasters (note that these estimates also include the damages caused by the 2010 Attabad landslide which destroyed parts of KH and new routes had to be carved inside the mountains).

Further analysis of the two timelines, 2003–2010 and 2011–2019, reveals that the prior time period is responsible for fewer number of fatal landslides, primarily because landslides were less frequent. In that same period, we see that rainfall-triggered fatal landslides were responsible for over 61% of the fatal landslides, whereas in the second period, the fatal landslides triggered by rainfalls accounted for only over 32%, which is a sharp decrease. However, the main triggers in the second period were construction and spontaneous events, which collectively saw an increase of 33.91% compared to the first time period. In Pakistan, earthquakes were involved in 3.33% of the fatal landslides (excluding 8 October 2005 earthquake event), and in the first time period, they caused fatal landslides which accounted for 6.78%, whereas in the second period, it was only 1.65%. Furthermore, by analyzing the monthly data, December is the month that records the least number of landslides, but the least proportion of fatal landslides was observed in the month of June that had a share of just 3.3%.

While it has been observed that in the time period from 2011 to 2019 the main causes of fatal landslides were spontaneous and due to construction or mining along the slopes, it cannot be overruled that the rainfalls were not significant. As many as 60.53% of all the landslides in this period were caused by rainfall (rainfall and heavy rainfall) events, which is a significant percentage. The reduction in the fatal landslides in a rainy season might be due to an increasing awareness in the mountainous communities, but on the other hand, spontaneous landslide events are even more alarming and need to be studied with geophysical tools (Table 2).

**Table 2:** Distribution of landslides and fatal landslides according to months; these statistics are based on 204 months of data

Month	Landslides	% landslides	Fatal landslides	Fatalities
JAN	47	4.31%	16	89
FEB	55	5.05%	20	95
MAR	48	4.41%	19	101
APR	291	26.72%	32	252
MAY	204	18.73%	8	35
JUN	55	5.05%	6	26
JUL	154	14.14%	21	150
AUG	58	5.33%	14	132
SEP	56	5.14%	15	51
OCT	81	7.44%	13	71
NOV	25	2.29%	8	44
DEC	15	1.38%	8	26

Pakistan is a very active seismic region and has seen multiple high magnitude earthquakes in the near past, 2005, 2010, 2013, 2015, 2017, etc. However, some scientific studies claim that the earthquake in 2005 could have killed more than 25,000 people in Azad Jammu and Kashmir (AJK) which generated as many as 2500 landslides (Dunning et al. 2007; Mahmood et al. 2015), but it could not be verified which of the specific regions had recorded what number of landslides (fatal and nonfatal) and casualties (note that the only landslides that were verifiable with date, time, damages, and, more importantly, geolocation were included in our study). Other landslide events caused by earthquakes within the 17 years of study timeline accounted for 10.19%; however, they were less deadly compared to other triggers in our study. In the period from 2003 to 2010, deadly landslides due to earthquakes had a proportion of 6.77%, and in the last period of our study, it was only 1.65%.

The increase in landslide events in Pakistan is related to extreme weather phenomena as the effects of climate change are already affecting the weather cycles and Pakistan is among the countries most effected by the climate change; it suffered economic losses worth 3.8b$ from 1999 to 2018 (Deccan-Herald 2019, Eckstein et al. 2019). Our study finds that rainfall and heavy rainfall events, which have increased in the recent years, are the major cause of trigger for the fatal landslides in Pakistan, and they are also the main factor in deadly landslides in a study conducted for 128 countries worldwide (Haque et al. 2016, 2019). However, our study also finds that the second main trigger for the fatal landslides in Pakistan is the occurrence of spontaneous landslide events, which is of greater concern. The landslide hotspots are mapped according to the districts of Pakistan (Fig. 7 a–c).

## Discussion

There is significant literature available on the role of climate change on the occurrence of landslides, whereas our study focused specifically on the occurrences of landslide-related events and their main triggers. Nevertheless, it is hard to rule out the impact of changes in the climate on the weather cycles. For the Himalayan plateau, which consisted of frozen solid ice a few hundred years ago, globalization and mass industrialization that increased the levels of CO_2_ in the atmosphere have had catastrophic effects on the health of mountains environment. The glaciers receded, and ice melted which has left the mountains in the northern regions of Pakistan with no vegetation, lose rock, and with the rock decay due to rainfall and other climate variables making the slopes of mountains highly vulnerable and risky.

**Fig. 8:**
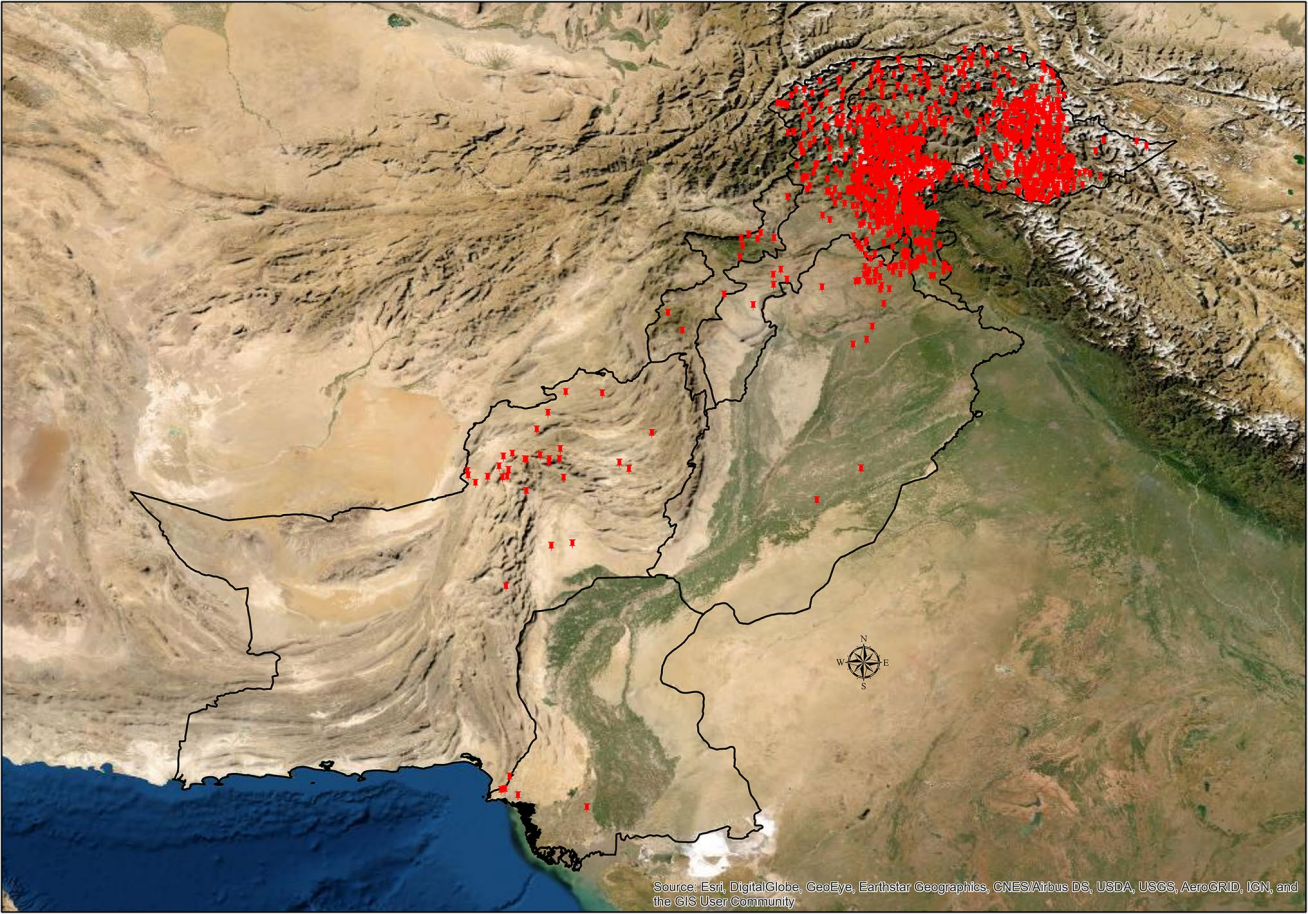
All the landslides observed in our study period 2003–2019

**Fig. 9:**
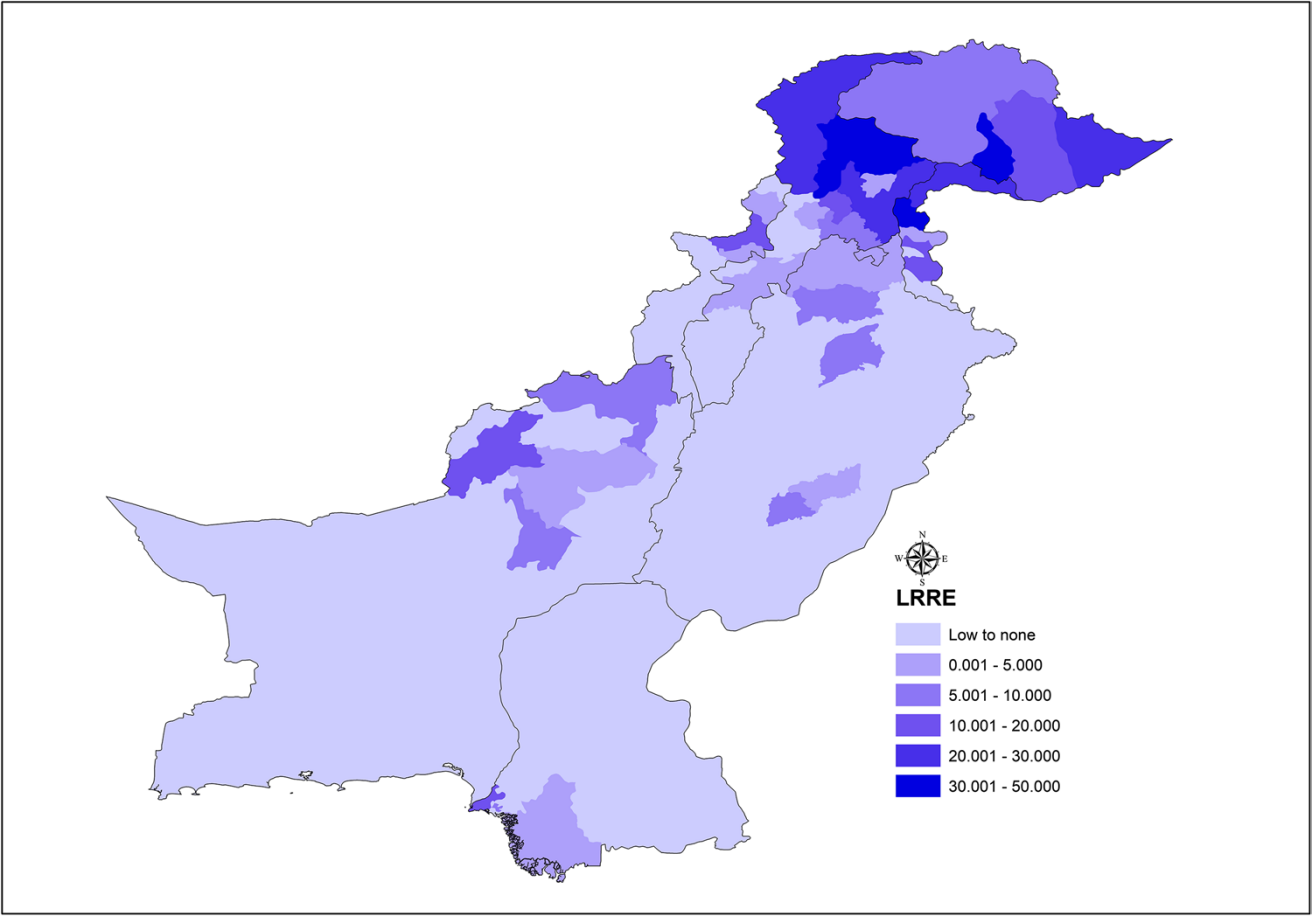
Distribution of landslide regional risk estimator (LRRE)

**Fig. 10:**
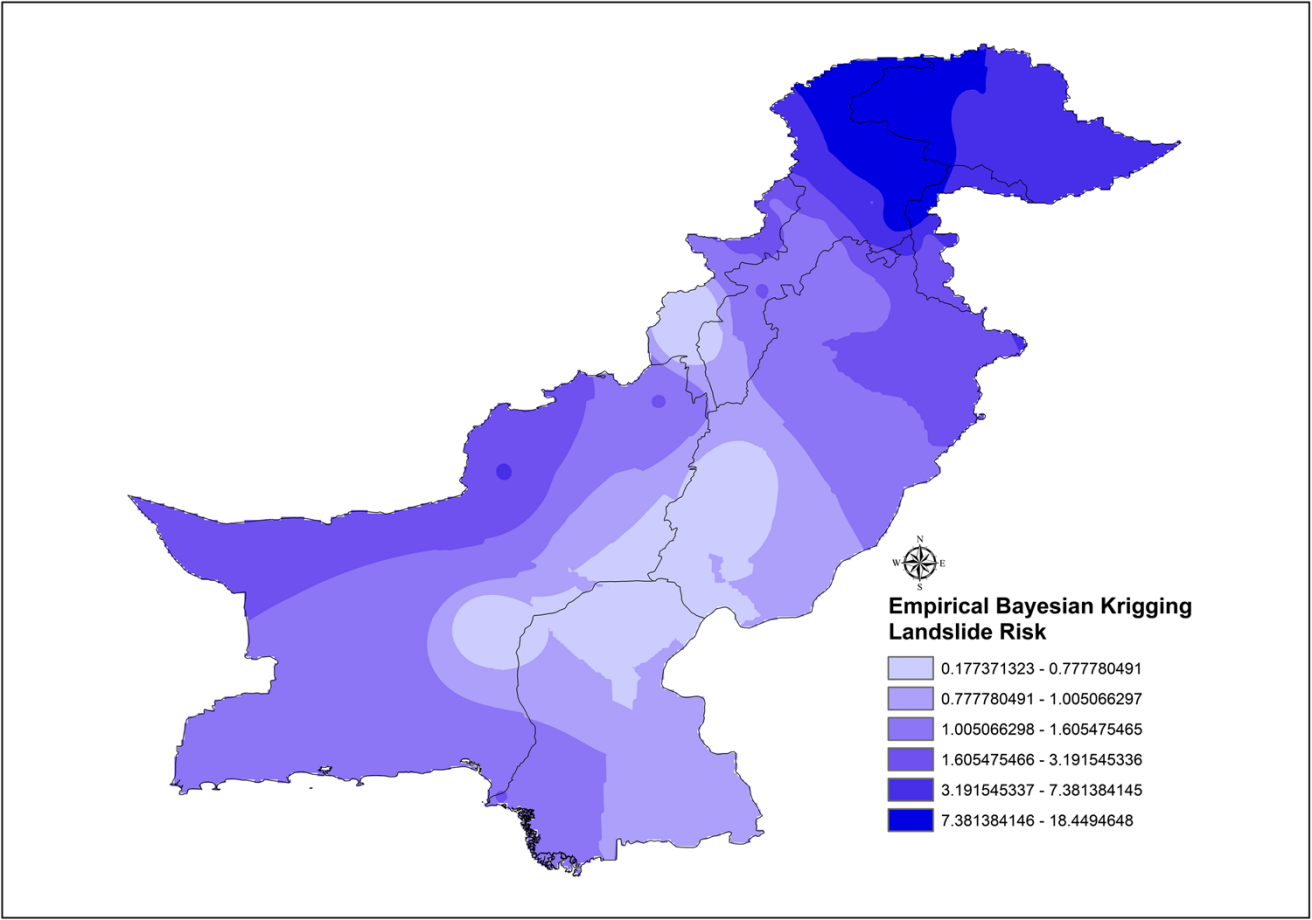
Empirical Bayesian Kriging (EBK)

There were several of the landslides or rockfall events in our study which were related to roads blocked due to rockfalls or heavy boulders falling on the roads, especially in the Northern parts of KH in Pakistan. On the other hand, most landslides were caused by construction, mining, and digging along the steep slopes in the province of Balochistan, which has the smallest population and population density in Pakistan. The province of Sindh is not particularly mountainous, but there are few hilly regions in the south-western Sindh. In these regions, the incidences of landslides have been attributed towards mining the clay and making houses on/under such steep slopes that can trigger mudslides. For instance, in an event of heavy rainfalls, such events have been recorded in Karachi, in the area known as Kati Pahari (lacerated hill). The province of Punjab has also seen fewer incidents of landslides because a vast majority of the population lives along the riverbanks. In the hilly terrains, and in the north-eastern part, where not a very large population lives, an exception is the district of Rawalpindi that includes the region of Murree, which is mainly mountainous. In the recent past, many landslides and landmass movements have been recorded mainly due to rainfall events that every so often generate floodings which further increases the chances of a landslide or debris flow by weakening the bases of the slopes or cause erosion (Figs. 8, 9, and 10).

The real-time effect of landslides in the districts is of greater interest. The district of Diamer recorded 217 landslides, whereas the district of Swat had only 14 landslides, but the fatalities in Swat were higher (due to the majority of population living alongside potentially unstable mountain slopes) than those of the district of Diamer (almost twice as many). Similarly, the data showed that the chances of fatalities (due to events of landslides) in Skardu were 3.5 times more than in the district of Mansehra. Most deaths were recorded in Kohistan with 17% of all the fatalities in our study period, followed up by Muzaffarabad with 10.7%, and Baltistan with 9.2%. The potential risks can be analyzed in more detail using the landslide regional risk estimator (LRRE, given in Eq. 1) which utilizes the information of the occurrences of landslides and casualties (regional based) from all the districts in the study. The estimator talks about the potential danger levels (chance of casualties) of individual districts in case of landslide incidences (Table 3).

**Table 3:** Distribution of occurrences of landslides and LRRE (54 districts were observed from 2003 to 2019)

District	Average precipitation mm (yearly)	Landslides	Fatal landslides	Casualties	LRRE
Swat	894	14	08	74	0.3578
Mansehra	1194	83	12	83	0.2304
Abbottabad	1262	17	08	36	0.2390
Shangla	1040	28	07	45	0.2164
Rawalpindi (Murree)	1347	30	02	02	0.0238
Chitral	414	28	06	54	0.2195
Skardu	202	17	07	141	0.4425
Muzaffarabad	1457	49	14	150	0.4449
Dir	120	13	05	39	0.2084
Neelam Valley	1350	19	05	60	0.2372
Poonch	1225	07	03	41	0.1818
Gilgit	133	69	03	16	0.0563
Kohistan	648	158	21	250	0.3417
Baltistan	150	216	08	108	0.1088
Diamer	500	217	10	67	0.0955

1 $$\mathrm{LRRE }=\frac{{\left\{\left(\theta +\xi \right)*\psi \right\}}^\frac{1}{2}}{\Sigma +\Omega }$$

In Eq. 1, $\theta$ is the count of fatalities, $\xi$ represents the number of injuries, and $\psi$ is the total number of fatal landslides in a region of interest. $\Sigma$ represents the total number of occurred landslides, and $\Omega$ is the total number of districts involved in our study.

The data was analyzed using the Empirical Bayesian Kriging methodology which further constitutes the fact that the results using LRRE are approximately similar in the risks prevalent in the districts across Pakistan. LRRE estimates the potential risk to an area by encompassing the factors directly associated with the severity of landslides as is mentioned in Eq. 1. Kriging predictors are generally used because the prediction error is minimized, but the Empirical Bayesian Kriging accounts for additional error introduced by estimating the semivariogram model (Pilz and Spöck 2008; Krivoruchko 2012). The main advantages of the Empirical Bayesian Kriging include nominal use of experts’ knowledge via collaborative modeling; it provides more accurate measurements of the standard errors of prediction, and also it is more accurate than the other Kriging methods for smaller sample-sized data. The general drawbacks include slower processing time especially when it is being output to raster, and log empirical transformation is sensitive to the outliers. There is a high probability that with any increase in the input points or subset size, the processing will get intensive and take longer to output.

Northern Pakistan, also known as Gilgit-Baltistan, is one of the most beautiful sites in the world with its tall mountains, many of them above 6000 m in altitude. This part of Himalayan system was once covered by solid ice, and the glaciers walls stood high between civilization and the mountains. The situation nowadays is on the contrary as the glaciers have receded and ice has melted from the mountains, which has exposed the lose rock which was once trapped in the ice. The region of Gilgit-Baltistan has recorded (only among the known or recorded cases) the greatest number of landslides, and yet due to it being a remote region, there is very little information available on the actual impact of landslide events because many of small rockfalls and landslides are cleared by the locals, and it does not get recorded.

Attabad in Hunza valley saw a catastrophe in 2010 when a huge landslide blocked the whole river Hunza and has created an artificial lake. However, thousands of people had to leave after their homes were submerged by the rising water levels; unfortunately, fatalities were also reported due to this event. There are not enough safety measures taken by the authorities to overcome the issues of rockfalls and landslides; one main reason is the difficult terrain along KH. There are few preventive measures that can be implemented, for example, an early warning system can be installed where the Disaster Management Authority (DMA) can send emergency text messages to the local residents of the regions in Gilgit-Baltistan to avoid any unnecessary traveling on KH or any link roads that are and will be further potentially dangerous in case of a rainfall event, according to the forecast and during a developing weather phenomena that can generate a downpour and hence make the slopes susceptible. It has been pragmatic that the incidences of landslides are on the rise globally, and this study further ascertains that such occurrences are also on the rise in Pakistan (Gariano and Guzzetti 2016; Petrucci 2022). As in multiple other research articles, we have observed and concluded that the risk of a landslide (fatal or nonfatal) is increasing, in Pakistan as well, substantially. Interestingly, we have observed slight reduction in the number of fatal landslides due to rainfall events, compared to global trends (Haque et al. 2019), as they account for only one-third of all the triggers of fatal landslides. Furthermore, there is a need for better housing structures, and around the vulnerable villages, there could be a preventive wall that can take the direct impact of a landslide, and this would reduce the damage to greater extent.

## Conclusion and suggestions

This study found that, in the period 2011–2019, there has been an increase in the total number of landslides, and it is also responsible for the highest number of fatal landslides, compared to 2003–2010. The hotspot regions for the landslide events are in Gilgit-Baltistan, Kohistan, Diamer region, and Muzaffarabad region in AJK. Furthermore, the post spring season is the deadliest due to landslides when the snow melting begins and rainy season starts. Mining and construction have been a major trigger in the second time period of our study, with a rise of more than 20% of the fatalities compared to 2003–2010. Fatal landslides due to spontaneous events were responsible for one-third of the deaths, and we have witnessed a big increase compared to the first time period of this study. However, rainfall and heavy rainfall were not the deadliest of the triggers in 2011–2019, but these events were responsible mainly in the first period and for the combined one (2003–2019). It is ascertained that the rainfall-related events are the biggest cause of triggering the landslide events in Pakistan. We suggest that an early warning system (reacting to critical weather forecasts) should be installed in regions which are more susceptible to landslides and rockfalls. Investments can also be put in fields like prevention and training the respective departments to efficiently deal with a post-landslide situation. This paper has identified the regions in Pakistan with the highest vulnerabilities, and we believe that the analysis presented in this paper will motivate others to carry out further analysis of the role of climate change; in particular, temperature increases/decreases along with historic dynamics of rainfall, floods, seismic activities, etc.

## Data Availability

The datasets used in the study are available upon request.
